# Eco-friendly adsorbents based on abietic acid, boswellic acid, and chitosan/magnetite for removing waste oil from the surface of the water

**DOI:** 10.1007/s11356-022-20169-2

**Published:** 2022-04-26

**Authors:** Mohamed Fekry, Salwa M. Elmesallamy, Nasser R. Abd El-Rahman, Mahmoud Bekhit, Hend Alaidy Elsaied

**Affiliations:** 1 Polymer Lab, Petrochemical Department, Egyptian Petroleum Research Institute, Nasr City, Cairo Egypt; 2 Surfactant Lab, Petrochemical Department, Egyptian Petroleum Research Institute, Nasr City, Cairo Egypt

**Keywords:** Magnetite nanoparticles, Boswellic acid, Rosin, Chitosan, Petroleum oil removing

## Abstract

Petroleum oil leakage and industrial oily waste on the water surface are sustainable pollutions. The removal process by eco-friendly adsorbents is a critical challenge. It also requires sustainable treatment. The natural hydrophobic material such as abietic acid, boswellic acid, and chitosan was added to magnetite nanoparticles with different concentrations of 10, 15, and 20% on its surface. The magnetite acquires partially hydrophobic properties. The prepared natural adsorbents were analyzed by employing wide-angle X-ray diffraction (WAXD), vibrating sample magnetometer (VSM), Fourier transform infrared spectroscopy (FTIR), scanning electron microscope (SEM), particle size and zeta potential, and contact angle measurements. Chitosan adsorbs at the outer surface of magnetite nanoparticles while boswellic and abietic absorb in bulk. All prepared adsorbents are effective in adsorbing waste oil from the water surface. The contact angle of MB20 (magnetite/20 percent boswellic) is greater than that of MA20 and MC20 (magnetite/20% abietic or chitosan, respectively), indicating that it has more hydrophobic characteristics. The oil removal efficiency and adsorption capacity of MB20 are the highest values 57.6%, and 24 g/g, respectively. All eco-friendly adsorbents are nontoxic with low-cost production and are used many times.

## Introduction

Crude petroleum oil is one of the sources of non-renewable energy; it considers an essential material, but it contains polycyclic aromatic hydrocarbons (organic pollutants) that are highly toxic environmental (Ben Jmaa and Kallel 2019; Ejeromedoghene et al. 2020). The presence of pollutants for a long-time has negative impacts not only on the plants, animals, and marine organisms but also on human health. More than 5 million tons of crude oil is transported annually via the ocean (de Oliveira Soares et al. 2020). There are nine million barrels of crude oil leaks in water bodies each year via different processes such as oil drilling, extraction, transportation, refinement, and accidental leakage (Liu et al. 2021b). The essential techniques for cleaning the oil waste were based on physical and chemical techniques like skimmers, barriers, booms, dissolved air flotation, dispersant or surfactant spray, and synthetic or biomass-based adsorbents, whereas there are other techniques, such as biological, which depend on bioaugmentation and thermal by in situ burning of waste oil (Pete et al. 2021). The weather influences the structure and its rate of degradation (Madison et al. 2020). There are different types of renewable sorbents to clean up petroleum waste, such as starch, cellulose, chitosan, lignin, vegetable oil, and rosin acid (Negi and Singh 2020). The sorption process depends on the surface, internal structure, and pores of the sorbent (Zhu et al. 2017).

Rosin (abietic acid’s source) is a renewable material, and its source is available in nature. It comprises 10% neutral and 90% abietic acids with a rigid structure produced from hydrogenated phenanthrene ring; it is used as an alternative to petroleum-based upon cycloaliphatic or aromatic. The carboxyl group and double bond in abietic acid can be employed through crosslinking agents, addition, disproportionation, decarboxylation, polymerization, esterification, salt formation, and hydrogenation (Mandal et al. 2018). Rosin is used in various thermosetting resins, and it operates in adhesive, soaps, heat preservation materials, tackifiers, sealing wax, biomedical applications, paints, plasters, and varnishes (Foti et al. 2021). Their derivatives are used as feedstock in polymeric substances. The annual production is 1.2 million tons, and its cost is low (Zheng et al. 2010).

Boswellic acids (BAs) are pentacyclic triterpenoidal; it is produced from the oleo gum resin of the plant Boswellia species, which is found in India, Northern Africa, and the Middle East (Mehta et al. 2018). It mainly contains four constituents: α-boswellic acid, 11-keto-β-boswellic acid (KBA), β-boswellic acid, and 3-acetyl-11-keto-β-boswellic acid (AKBA) (Mehta et al. 2016). BA has been reported to have significant pharmacological properties such as anti-inflammatory activity. BA is also utilized in arthritic pain, colitis, antihyperlipidemic, antifungal, antibacterial, and anticancer (Iram et al. 2017).

**Fig. 1:**
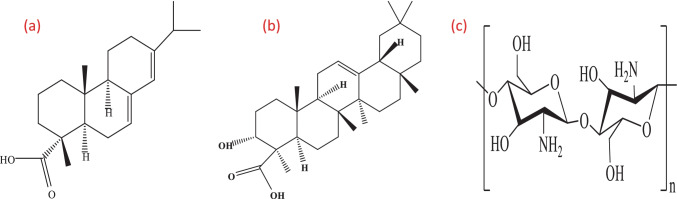
Chemical structure of **a**) abietic acid, **b**) boswellic acid, **c**) chitosan

Chitosan is a biopolymer obtains via the deacetylation of chitin; it is extracted from marine sources. It can adsorb pollutants such as heavy metals, dyes, pharmaceutical residues, and waste water. It has a distinctive structure with higher hydrophilicity and low surface area, decreasing the oil adsorption capacity. So, there are different processes for functionalization of chitosan to improve its adsorption capacity, such as grafting, composite formation, and crosslinking (Desbrières and Guibal 2018). Oil adsorption mechanism depends on opposite charges and interlace effect between oil and chitosan (Negi et al. 2021). The chemical structure of abietic acid, boswellic acid, and chitosan is shown in Fig. 1.

This work aims to synthesize natural magnetic sorbent with unique properties such as biodegradable, reliable, and available in nature and low-cost production, high surface area, eco-friendly, low toxicity, easily recycled, and disposable to remove waste petroleum oil from the water surface.

## Experiment

### Materials

Ferric chloride, FeCl_3_·7H_2_O (M.W 270.3), was purchased from Nice Co., India. Ferrous sulfate, FeSO_4_.7H_2_O (M.W 278), was purchased from Loba Chemi Co. India. Ethyl alcohol and sodium hydroxide pellet solid NaOH was obtained from El Nasr Pharmaceutical Chemicals Co, Egypt. Rosin and Frankincense were commercially purchased. The paraffinic crude oil was supplied from Qarun Company (Egypt), and the characteristic and properties of waxy crude is tested as follows: API gravity at 60 °F is 32.2, specific gravity is 0.86 kg/m^3^, wax content is 11.4 wt %, asphaltene content is 1.3 wt %, and pour point is 28 °C.

### Characterizations

#### FT-IR

The spectra were registered through the spectrometer model type Mattson-Infinity Series Benchtop 961 and have been employed; 1 mg of each sample of raw and modified alumina silicate was ground and specified in the range of 4000–400 cm^−1^ with a resolution of 2 cm^−1^.

#### Particle size and zeta potential

The particle size distributions and zeta potential were measured with Malvern Zeta sizer nano series (NANO-ZS) HT using dynamic light scattering in ethanol.

#### Wide angle X-ray diffraction

Wide angle X-ray diffraction (WAXD) was registered with a Pan Analytical Model X Pert Pro, which was connected with CuK a radiation (*k* = 0.1542 nm), Ni-filter, and a general area detector. The diffract grams were recorded in the 2-h range of 10–40 °C with a step size of 0.02°A and a step time of 0.7000 s, with generator settings 40 MA, 40 kV.

#### VSM

Magnetic measurements were executed with the Quantum Design Model 6000, and parameters such as (Ms), (Hc), and (Mr) were evaluated.

#### Contact angle

The contact angles were measured for MB15, MA15, MC15, and M. All previous materials were pressed in a template with a 1-cm-diameter at the same pressure and room temperature of 25 °C. The contact angles were tested on the pressed surface. The contact angles were recorded for 15 s.

#### FE-SEM

The surface morphology for MB15, MR15, MC15, and M was detected by using ZEISS (FEI Quanta FEG 250).

#### Preparation of magnetite nanoparticles

The magnetite nanoparticles were synthesized using a co-precipitation method; they were synthesized by mixing ferric chloride FeCl_3_·7H_2_O and FeSO_4_·7H_2_O with ratio (1:2) in deionized water through a three-neck flask. The nitrogen purged was connected with the solution. NaOH solution was added drop-wise into the previous solution. The final pH was adjusted to 9. The temperature was raised to 90 °C for 3 h. The byproduct (salts) was removed from the precipitated nanoparticles by rinsing with deionized water four times. The final pH was decreased to 8.5. The precipitated nanoparticles were dried in an oven at 60 °C for 2 h (Fekry et al. 2021).

#### Preparation of chitosan

The deproteinization of shrimp shells occurred using NaOH solution 3.5% (w/w) for 3 h at 70 °C; then, the mixture was demineralized by 1 N HCl for 1 day. Acetone was used as a removing color for 2 h at 50 °C and dried for 4 h. The acetyl groups were removed from the chitin by mixing NaOH (55%) for 3 h at 120 °C with a ratio 1:10 (w/v). The resulting chitosan was neutralized with water, filtered, and dried at 70 °C. The degree of deacetylation is 92% (Hussein et al. 2013).

#### Preparation of magnetite/abietic acid and boswellic acid nano-composites

The magnetite/abietic acid 10% nanocomposite (MA10) was prepared by the casting method. The abietic acid was dissolved in ethyl alcohol with a ratio (1:10) (w/v). After the complete dissolving, the prepared solution was cast on magnetite powder with a ratio (100:100) (v/w). The mixture was strongly mixed and dried at 60 °C in an oven. The produced powder was crushed. MA15 and MA20 were prepared with the same previous method but with ratios 150:100 and 200:100, respectively. The magnetite/boswellic acid 10, 15, and 20% nanocomposite (MB10, MB15, and MB20, respectively) was prepared by the same previous method.

#### Preparation of magnetite/chitosan nanocomposites

Chitosan solution (2% wt/v) was designed in aqueous acetic acid (1% v/v), the mixture was homogenized by a magnetic stirrer for 1.5 h, and the previous mixture was filtrated. Magnetite nanoparticles were prepared by mixing ferrous sulfate and ferric chloride with a molar ratio 1:2, and then added to an aqueous solution of NaOH drop by drop to induce the chemical co-precipitation was heated at 80 °C for 2 h; the final pH was kept at 8. Finally, magnetite was washed several times with distilled water. The chitosan solution with different concentrations of 10, 15, and 20% was mixed with magnetite solution for 2 h using a strong mechanical stirrer; tripolyphosphate solution was added drop by drop until pH 8. The prepared nanoparticles (MC10, MC15, and MC20) were filtrated and dried at 60 °C for 6 h. The produced powder was crushed (Wan et al. 2018).

### Oil sorption capacity and removal oil efficiency

The oil sorption test was classified as type II with 10 min (short test), contact time of at least 4 g of oil, and sorbents having an unconsolidated particulate form. Oil sorption from the water surface was determined by forming an oil film on the water surface without shaking. The removing ability and oil sorption capacity of pure M, MB, MR, and MC nanocomposites with different concentrations of 10, 15, and 20% were measured at 25 °C. The pour point of waste oil is 27 °C. All the prepared adsorbents are solid powder; they were sprayed on the surface of the oil. After a certain time, the adsorbed oil on the surface of the magnetic adsorbent was taken off by using a NdFeB magnet (5000 Gs). The removal efficiency was counted by Eq. (1):

1 $$\mathrm{REF}\left(\%\right)=\left\{\left(C_0-C_r\right)/C_0\right\}\times100$$

where *C*_0_ is the initial weight of waste oil, and *C*_*r*_ is the weight of residual oil content. Each sample was tested three times, and the reported REF value is the average value of three test results. Oil adsorption capacity (ODC) for nano-adsorption was calculated by the Eq. (2):

2 $$\mathrm{ODC }= \left({C}_{0} - {C}_{r}\right)/{C}_{A}$$

where *C*_*A*_ is the weight of the nano-adsorbent; this equation indicated the number of oil adsorption. It was measured the residual oil content after adsorbing on a magnet surface. A uniform distribution of adsorbent powder was obtained through a sprayer (Abu Bakar et al. 2021).

#### Wide-angle X-ray diffraction

X-ray diffraction analysis has been performed to estimate the particles size of M, MB15, MR15, and MC15% (solid powder) from the Scherrer formula Eq. (3):

3 $$D=\frac{0.9\lambda }{\beta \mathrm{cos}\theta }$$

where “${\varvec{\lambda}}$” is the wavelength of X-ray (0.1541 nm), “*β*” is FWHM (full width at half maximum) was measured in radians, “$\theta$” is the diffraction angle, and “*D*” is average particle size diameter (He et al. 2018).

#### Adsorbent dosage

The sorbent dosage was in the range of 0.1 g; the crude oil was in the range of 5 g. The oil adsorption capacity was evaluated in a static state without shaking.

#### Recycle and reusability test

The saturated adsorbent with oil was filtered in a vacuum and then dried at 70 °C for 12 h; finally, it was ground for the next recycle and reuse test.

## Results and discussion

### FT-IR analysis

**Fig. 2:**
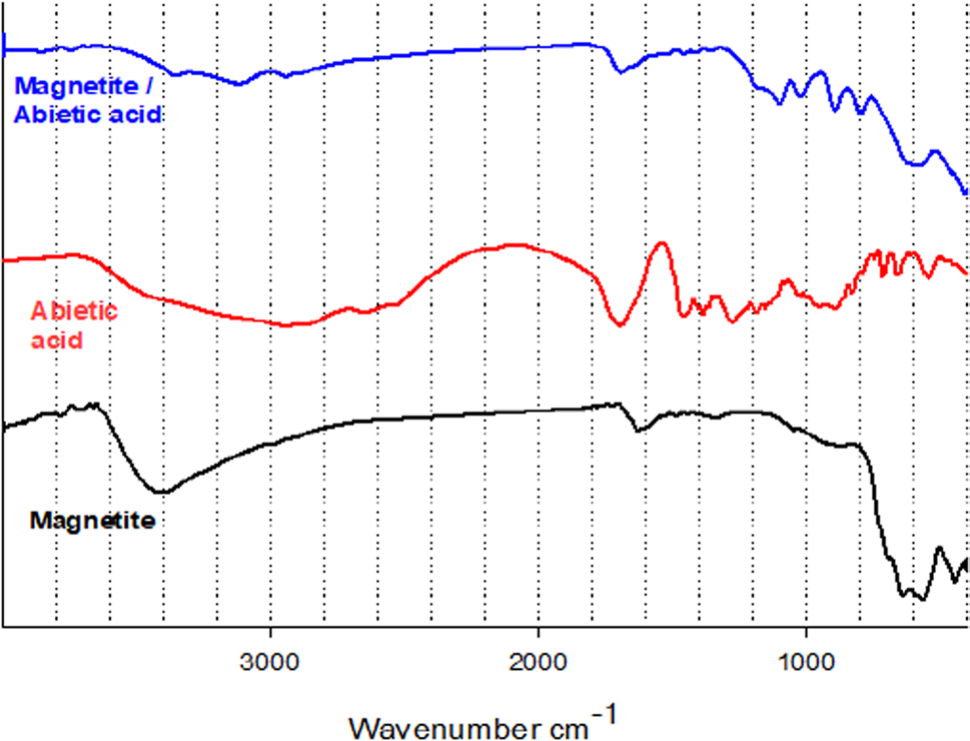
FTIR spectra of magnetite (M), abietic acid (A) and magnetite with 15% abietic acid (MA15)

**Fig. 3:**
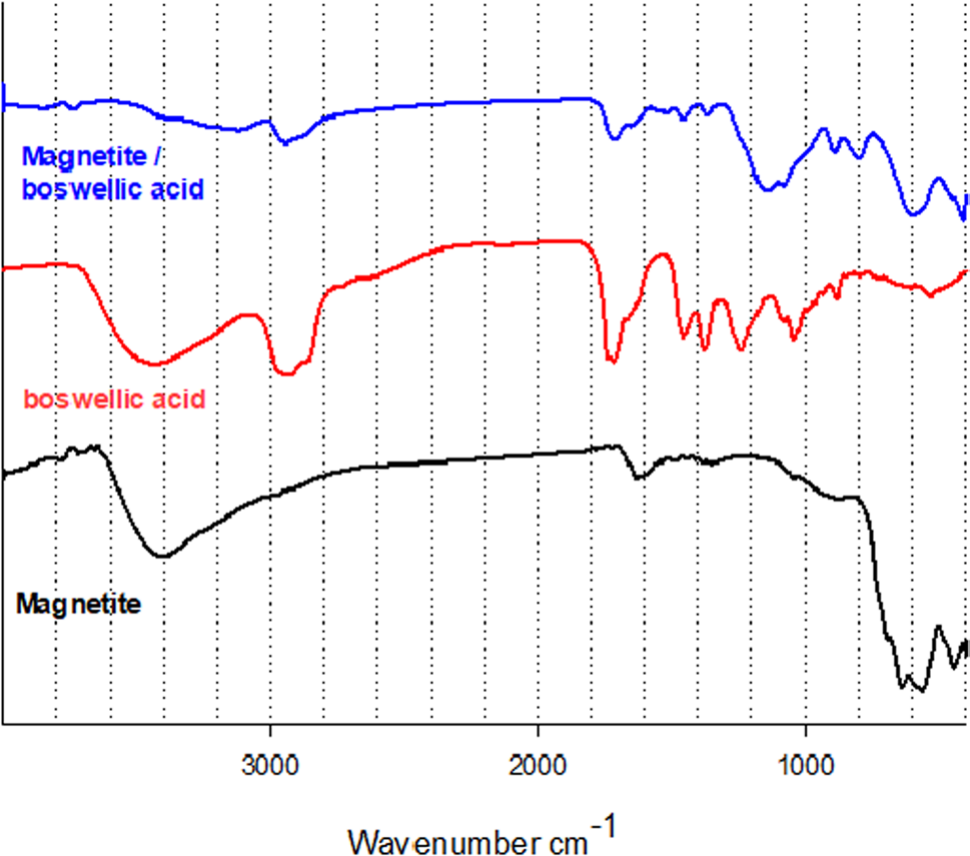
FTIR spectra of magnetite (M), boswellic (B) and magnetite with 15% boswellic acid (MB15)

**Fig. 4:**
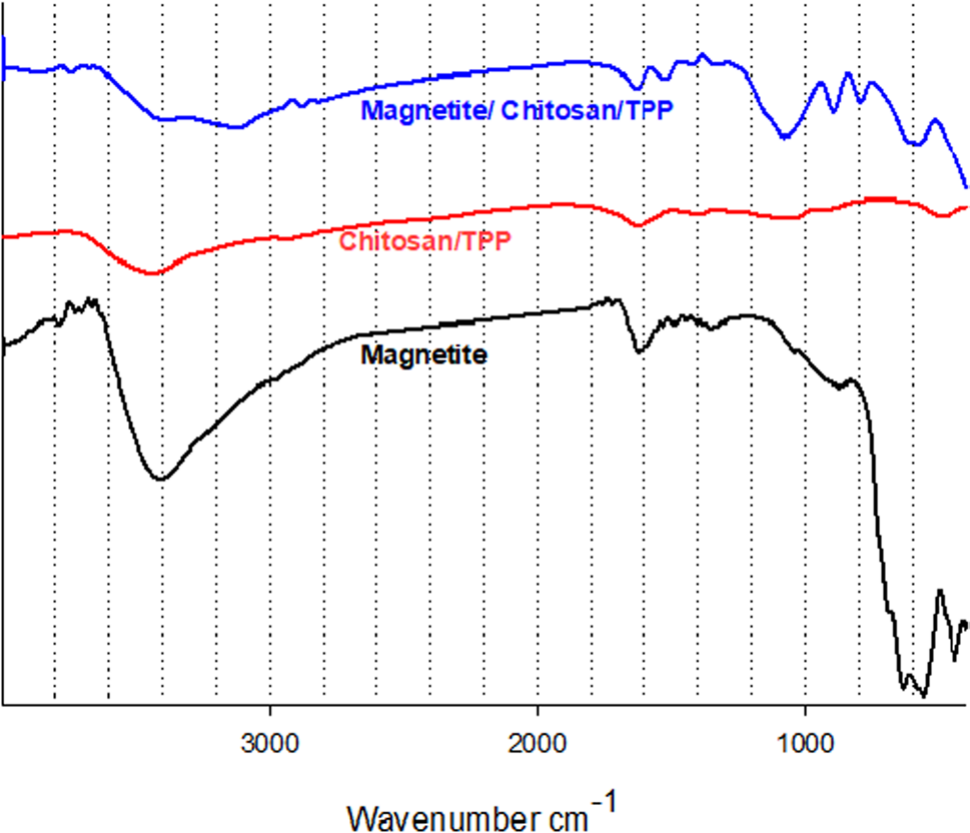
FTIR spectra of magnetite (M), chitosan (C) and magnetite with 15% chitosan (MC15)

The FTIR spectra of pure magnetite (M), abietic acid (A), and MA were investigated to detect their chemical structure as shown in Fig. 2. FTIR spectrum of magnetite nanoparticles is shown in Figs. 2–4. At the 638–563 cm^−1^ range, there are two absorption bands; these bands were attributed to the vibration of iron oxide bonds in tetrahedral and octahedral sites; they were produced from the split of the band nearly 570 cm^−1^. The band at nearly 440 cm^−1^ was attributed to the octahedral of iron only and corresponds to Fe–O of mass magnetite; it was shifted from the band at 370 cm^−1^. The broadband at 3408 cm^−1^ was attributed to O–H stretching vibration. The band at 1628 cm^−1^ was attributed to the existence of OH groups or water molecules. FTIR spectrum confirms that the magnetite nanoparticles absorb the added abietic acid (Mazrouaa et al. 2019).

The FTIR spectrum of the abietic acid was studied. The absorption band between 2500 and 3000 cm^−1^ refers to the strong stretching vibration OH, which refers to the carboxyl acid dimer. There are some moisture observed. The peaks at 2545 and 2650 cm^−1^ refer to the stretching carboxyl group. The bands at 1459 and 1385 cm^−1^ refer to CH_2_ and CH_3_ bending. Abietic acid has three-ring structures in the diterpenes which give it the strong stretching vibration C–H of the methyl and methylene group at 2880 and 2935 cm^−1^. The sharp band at 1694 cm^−1^ assigns to stretching vibration (C = O) of carboxylic acid. Characteristic bands nearly 789 and 831 cm^−1^ refer to aromatic groups that appear, and the band at 1280 cm^−1^ refers to the O–H bending (Favvas et al. 2015). FTIR spectra of adsorbed abietic acid on the surface of magnetite nanoparticles were studied. The characteristic bands between the region 3100–3640 cm^−1^ refer to the anti-symmetrical and symmetrical stretching modes of water (contains moisture) or traces of ethyl alcohol which appear at 3120 and 3421 cm^−1^, respectively. Absorption bands of C–H of the methyl at 2880 and 2935 cm^−1^ still exist. The band at 1694 cm^−1^ was shifted to 1691 cm^−1^ due to the present small amount of iron hydroxide which interacts with the carboxylic acid group. Two absorption bands at 638–563 cm^−1^ still exist. The band at 444 cm^−1^ was disappeared. The FTIR spectra of pure magnetite (M), boswellic acid (B), and MB were investigated to detect their chemical structure as shown in Fig. 3. The FTIR spectrum of B shows the broad absorption bands at 3443 cm^−1^ correspond to O–H stretching vibration, the band at 2925 cm^−1^ assigns to C–H stretching vibration, the sharp band 1715 cm^−1^ attributes to C = O stretching of carboxylic acid, and the band at 1454 cm^−1^ is attributed to C–H bending. Moreover the peak at 1375 cm^−1^ attributes to − COO symmetric stretching of carboxylates, and the peak at 1240 cm^−1^ attributes to C − COC stretching of aryl ketone (Ranganathan et al. 2017). The FTIR spectrum of MB shows the newly appeared band at 3738 cm^−1^ due to bridging and bidentate coordination between the carboxyl group of boswellic acid and Fe atoms in magnetite. Thus, we conclude that iron boswellate was formed with low concentration according to low started concentration. Broad band O–H stretching at 3443 cm^−1^ was decreased, the stretching band at 2925 cm^−1^ assigned to C–H still exists, and the sharp stretching band C = O was shifted from 1715 to 1712 cm^−1^ due to the formation small percentage of salt between acid and hydroxide. The FTIR spectra of pure magnetite (M), chitosan/TPP (C), and MC were studied as shown in Fig. 4. From previous literature, the pure chitosan has characteristic absorption bands at 3428 cm^−1^ (O–H and N–H stretching vibrations), 2921 cm^−1^ (C–H stretching vibrations), 1650 cm^−1^ (N–H bending vibrations), 1073 cm^−1^ (C–O–C stretching vibrations), and the bands at 1320–1426 cm^−1^ (C–N stretching vibrations) (Liu et al. 2021a). The FTIR spectra of MC show a more flat and wider peak at 3360 cm^−1^ (O–H stretching) compared with the spectrum of chitosan, as a result of stronger hydrogen bonding. There are two peaks at 1647 and 1581 cm^−1^ of pure chitosan which are assigned to C = O stretching and bending vibration from the amide group and NH_2_ group shifted to 1634 and 1521 cm^−1^, respectively. This shift has occurred as a result of linkage between the amino groups in pure chitosan and phosphoric acid groups of tripolyphosphate. Two absorption bands of Fe–O at 638–563 cm^−1^ still exist.

### WAXD analysis

**Fig. 5:**
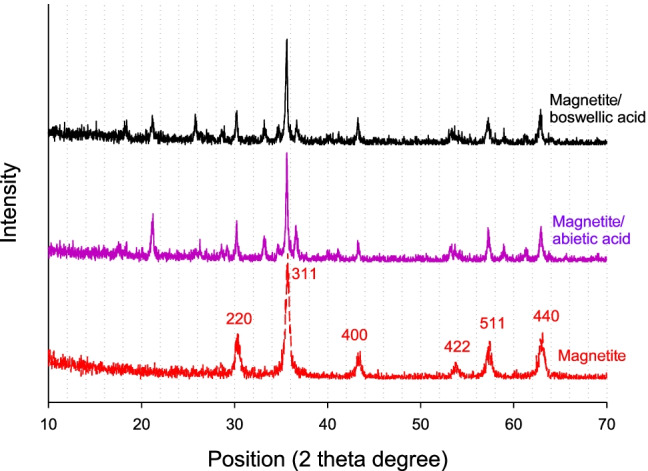
WXRD patterns of M, MB15, and MA 15

**Fig. 6:**
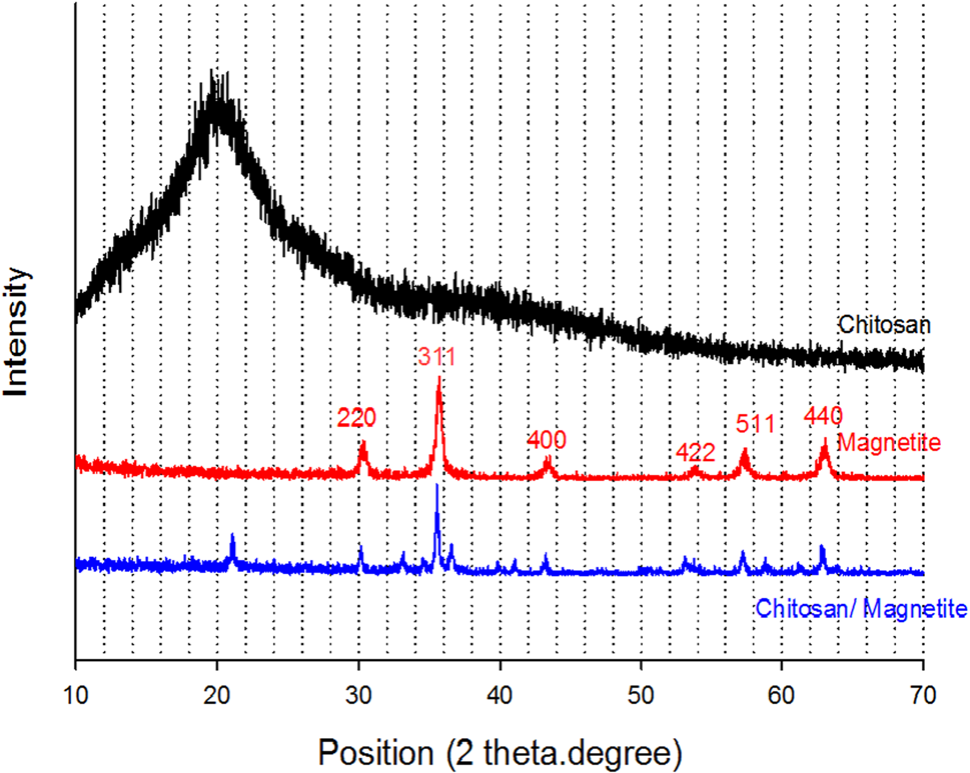
WXRD patterns of M, chitosan (C), and magnetite with 15% chitosan (MC15)

X-ray diffraction analyses have been executed to study the amorphous and crystallinity nature of the prepared nanocomposites MB and MA compared with magnetite nanoparticles as shown in Fig. 5. For pure magnetite, the main distinct peaks of the crystalline plane were assigned at 30.20°,35.58°, 43.2°, 53.5°, 57°, and 62.7°; they were matched with (220), (311), (400), (422), (511), and (440) respectively. The broadening of diffraction peaks is an indication of the size of magnetite nanoparticles. The produced peaks were similar to the standard magnetite diffraction pattern, and there are no changes in the crystalline nature of magnetite nanoparticles. The XRD patterns of pure abietic acid and boswellic acid from previous literature show the main broad characteristic diffraction peaks at (2θ) 15.66° and 14° respectively; they have amorphous structures (Jindal et al. 2016; Uthaman et al. 2012). The XRD patterns of MB and MA show the broad peak at 15.66°, and 14° disappeared; the main characteristic peaks of magnetite still exist. There are new peaks that appeared due to the oxidation of magnetite. A small percentage of maghemite (γ-Fe_2_O_3_) was formed during the mixing and drying of acid with iron oxide nanoparticles. The new peaks were at (2θ) 18.37°, 21.18°, 25.79°, 33.19°, and 35.6° (Zulfiqar et al. 2015). The XRD pattern of MB and MA explained that the magnetite nanoparticles absorb the acid in the bulk and do not adsorb on the surface. So, the amorphous structure of magnetite was not affected by the amorphous structures of both acids. The estimated crystal size diameter of the M, MB, and MA was 164, 30.4, and 34.8 nm, respectively. The obtained results explain the improvement in the values of particle size distributions. The XRD pattern of chitosan nanoparticles (C) and MC is shown in Fig. 6. X-ray diffraction characteristic peak of pure chitosan displays a broad peak at 2θ = 20°. The XRD patterns of MC have the main characteristic peaks of magnetite; it also has a weak peak at 2θ = 20° instead of a broad peak of chitosan. In addition, there are new peaks due to the oxidation of magnetite with acetic acid. The estimated crystal size diameter of the MC was 43.7 nm. The XRD pattern shows good compatibility between chitosan and magnetite; it also shows the adsorption of chitosan on the surface of magnetite as a core–shell structure. The obtained results from the Scherrer equation explain the average crystalline diameters. However, the actual size of nanoparticles does not equal the expected size from the equation, due to the agglomeration in a lattice structure, so the actual average diameters are large.

### VSM

**Fig. 7:**
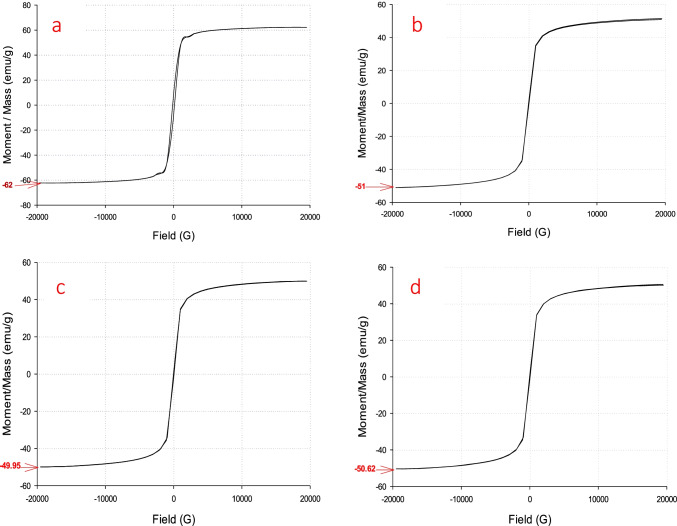
Hysteresis loops of M (**a**), MB15 (**b**), MA15 (**c**), and MC15 (d)

Magnetic properties play important roles in different applications. The hysteresis loop of all prepared adsorbents is shown in Fig. 7. The saturation magnetization (Ms) of M, MB15, MA15, and MC15 was 62, 51, 49.95, and 50.62 emu/g, respectively, at room temperature. The obtained data confirm that magnetite nanoparticles have superparamagnetic properties. The values of Ms for MB15, MA15, and MC15 nanocomposites are reduced compared with Ms for pure magnetite due to absorption and adsorption of the natural product on the bulk or surface of magnetite. The difference between the values of Ms for MB, MA, and MC is small. The values of coercive force and magnetic remanence of adsorbents have negligible values. So, the prepared adsorbents are strongly attracted to the magnetic fields to easily remove oil waste. The magnetization behavior of magnetic particles is proportional to their size. The smaller particle size leads to an increase in the ratio of surface-to-volume; consequently, the disorder of surface spin will increase, and the saturation magnetization will decrease. The obtained data agree with these facts; the particle size of prepared adsorbents is smaller than magnetite. The results show that the addition of magnetite with high superparamagnetic to the prepared natural product with diamagnetic properties improves the practical application of these materials in removing the petroleum waste from the water surface.

### Zeta potential analysis and DLS

**Fig. 8:**
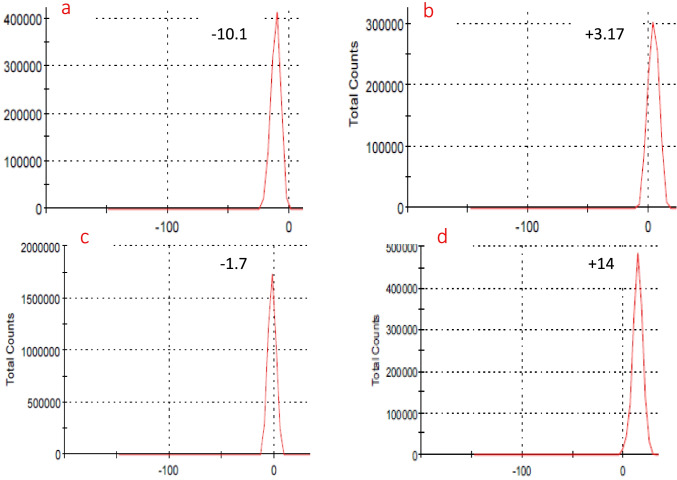
Zeta potential of M (**a**), MB15 (**b**), MA15 (**c**), and MC15 (d)

**Fig. 9:**
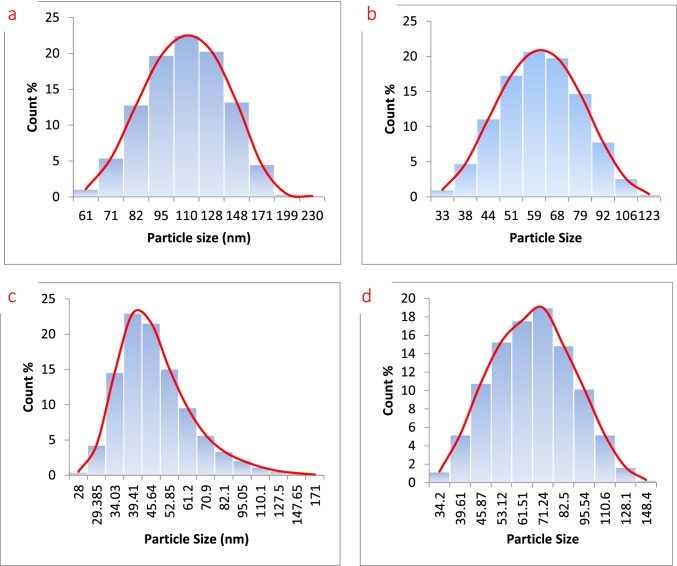
Particle size distribution of M (**a**), MB15 (**b**), MA15 (**c**), and MC15 (d)

The stability and dispersibility of particles in the solution are depending on the surface charges. The high value of charge (positive or negative) creates a repulsive force between particles and inhibits them from aggregation and precipitation. The particle size also affects the precipitation process. The zeta potential of M, MB15, MA15, and MC15 is shown in Fig. 8. The obtained data show zeta potential of the pure magnetite was increased from a negative value (− 11.2 to + 3.17, − 1.7, and 14 mV) due to the adsorption of boswellic, abietic acid, and chitosan respectively. The positive values of zeta potential were probably assigned to carboxylic acid and amine groups (Xu et al. 2019). There is electrostatic interaction between (OH) alkaline of magnetite and carboxylic and protonated amine. All the obtained values of zeta potential have small values lower than 30 mV. That means the prepared adsorbents are not stable in solution bulk. The stability and dispersibility of MC are higher than MB and MA. The particle size distribution of all prepared adsorbents is shown in Fig. 9. The average particle size of M, MB15, MA15, and MC15 was 130, 70, 68, and 79 nm, respectively. The detected average particle sizes of adsorbents are higher than those detected by XRD (Sherrer equation). The particle size of pure magnetite is larger than all prepared adsorbents due to the agglomeration of particles with each other. The particle sizes of all prepared adsorbents are small due to the influence of coating which makes them slightly rigid during the grinding process; the coating also inhibits aggregation. The prepared adsorbents in nano-size reveal that the amount of added materials such as boswellic, abietic acid, and chitosan are homogeneously distributed on the surface or the bulk of magnetite.

### SEM/EDS analyses

**Fig. 10:**
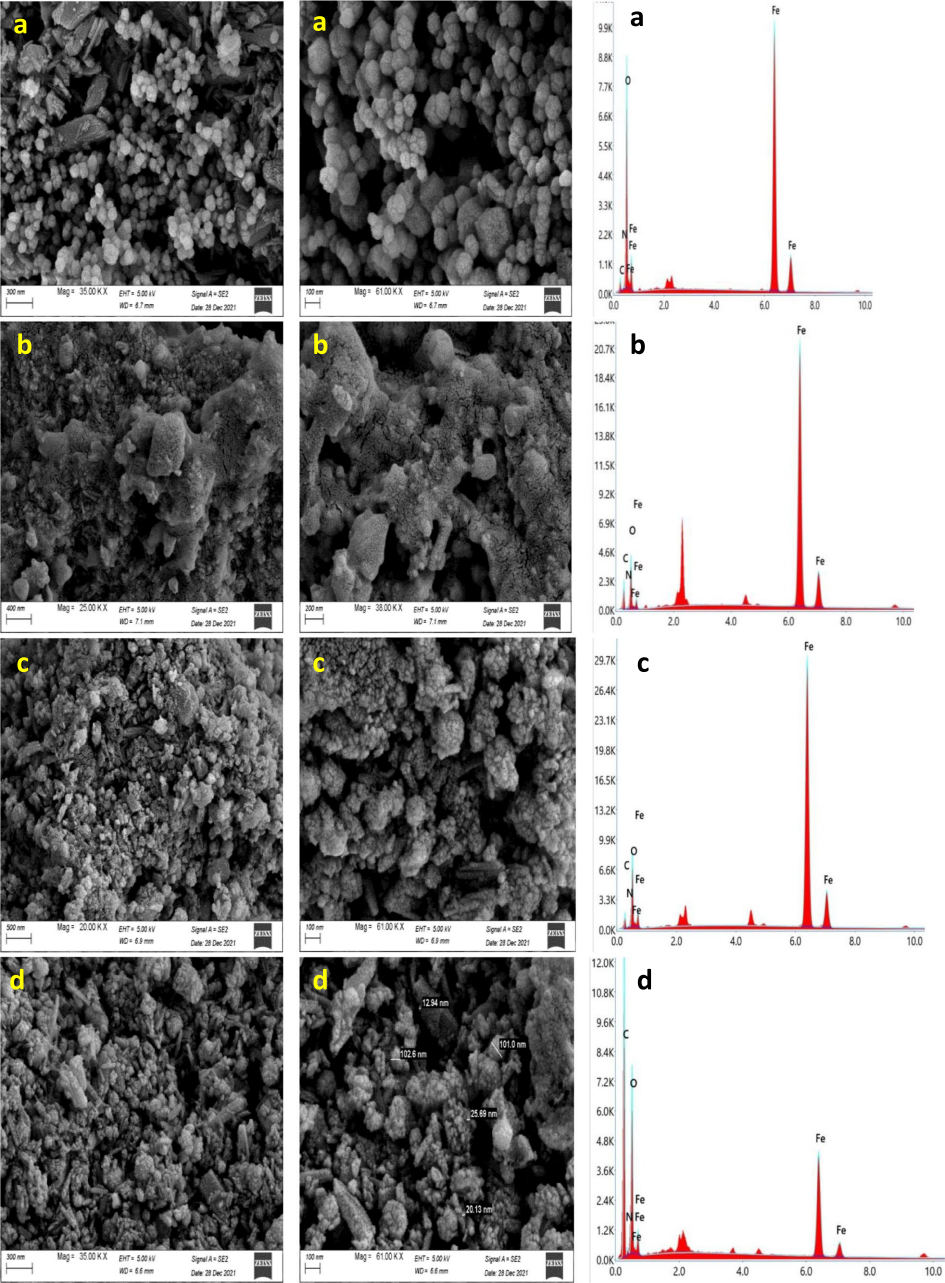
SEM images and their EDS of M (**a**), MB15 (**b**), MA15 (**c**), and MC15 (**d**) with two magnification

SEM images of M, MB15, MA15, and MC15 with high resolution and applied voltage are shown in Fig. 10. The morphology of magnetite is uniform distribution in nano-scale; it appears as spherical with high surface area and porosity, but there are small aggregations that produce large particles. The morphology of MB15, MA15, and MC15 illustrates that there is partial melting to boswellic and abietic acid on the surface of magnetite due to the high electron energy of cathode in the electron gun although it provides narrower probing beams; it causes charging at high magnification. The texture also illustrates a lot of clusters; each cluster contains a lot of particles in nano-scale with high porosity and small openings. It also shows the complete distribution of added materials on all surfaces of magnetite (Omidinasab et al. 2018).

The EDS spectra of M, MB15, MA15, and MC15 are shown in Fig. 10 and their elemental constituents in Table 1. The EDS spectrum of magnetite shows O and Fe atoms are the main constituents with 43.6 and 54 weight percentages, respectively. The EDS spectrum of MC15 illustrates a high weight percentage of carbon (51%) while Fe atom is 8% at the surface, which indicates the magnetite nanoparticles were surrounded by chitosan nanoparticles (El-kharrag et al. 2018). The EDS spectra of MB15 and MA15 contain low weight percentages of carbon (23 and 14% respectively); it indicates the magnetite nanoparticles absorb boswellic and abietic acid in the bulk of particles.

**Table 1:** Weight percentages of M, MB, MA, and MC from EDX data

Element	Weight % of M	Weight % of MB15	Weight % of MA15	Weight % of MC15
C K	1.5	23	14	51
O K	43.6	19.3	23.5	38
N K	0.5	0.45	0.7	4
FeK	54	57	62	8

### Removal oil efficiency

**Fig. 11:**
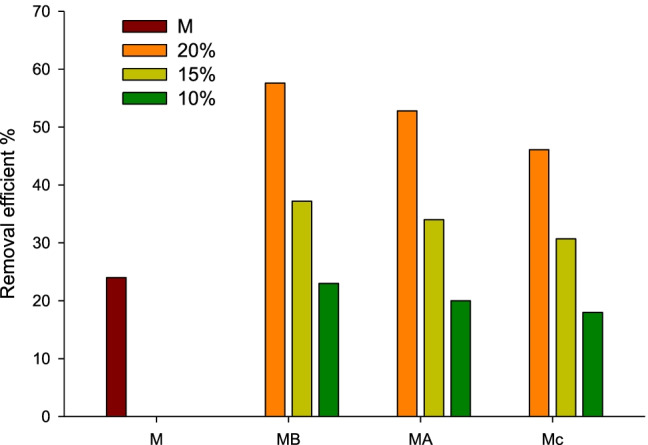
Removal efficiency of M, MB, MA, and MC at different concentrations 10, 15, and 20% at 25 °C

Offshore exploration, transportation, and oil production are still a great challenge. Marine oil pollution is sensitive to the growing risk from dispersant oil. The toxicity of the used chemicals also has bad effects on marine life. The oil spill was controlled by the eco-friendly dispersant. Moreover the dispersants in nano-size dimensions have many characteristics. The dispersants remain in the water to disperse the oil into tiny droplets (Badmus et al. 2021), but the adsorbents remove the oil droplets. Magnetite nanoparticles have been used in different applications such as drug delivery and medicine due to their unique properties; they have antimicrobial effects. The selectivity of oil/water by using absorbent is a recent promising development. Oil adsorbents should have superhydrophobic properties. The oil REF of M, MB, MA, and MC at different concentrations 10, 15, and 20% at 25 °C is shown in Fig. 11. All the prepared adsorbent powders were uniformly sprayed on the water surface to obtain a uniform distribution without agglomeration. In the case of pure magnetite, it immediately settles down in the bottom at once when it sprays on the water surface, but the prepared nano-adsorbent powder stays on the water surface for a long time without settling down. The magnetite nanoparticles adsorb the natural products to acquire hydrophobic character and protect it from oxidation. So, the prepared adsorbents have high selectivity to oil than water. After the adsorption process, the waste oil collects with a permanent magnet from the water surface. The oil REF of MB 20, 15, and 10% was 57.6, 37.2, and 24 respectively, which are higher than MR 20, 15, and 10% (52.8, 34, and 20 respectively). The oil REF of MC 20, 15, and 10% was 46.1, 30.7, and 18 respectively, which considers the lowest values with comparing with MB and MR. Generally, the coated magnetite with 20% is the highest oil REF value. The coated magnetite with only 10% is the lowest oil REF value. Table 2 shows the ODC and REF data for all prepared nano-adsorbent. The ODC of MB 20, 15, and 10% was 24, 13.5, and 8.5 g/g respectively; they consider the highest values with comparing with M, MA, and MC due to the adsorption of boswellic acid in the bulk of magnetite. The boswellic acid has a strong hydrophobic character, so the MB nanoparticles acquire partial hydrophobic character which depends on the percentage of added boswellic acid. The ODC of MA 20, 15, and 10% was 20, 12.1, and 7.14 g/g in comparison with ODC of MC 20, 15, and 10% that was 13.4, 8.88, and 5.2 g/g, respectively. The oil adsorption capacity of MC was the lowest value due to the coating of chitosan nanoparticles on the surface of magnetite nanoparticles which obstructs the oil adsorption of magnetite.

**Table 2:** Removal efficiency and oil adsorption capacity of all prepared adsorbents powder

Adsorbent	Oil waste adsorption (g)	Number of adsorption(g/g) (ODC)	Removal efficient % (REF)
M	1.3	5.9	24
MB20	2.6	24	57.6
MB15	1.9	13.5	37.2
MB10	1.2	8.5	23
MA20	2.8	20	52.8
MA15	1.7	12.1	34
MA10	1	7.14	20
MC20	2.4	13.4	46.1
MC15	1.6	8.88	30.7
MC10	0.9	5.2	18

### Contact angle

**Fig. 12:**
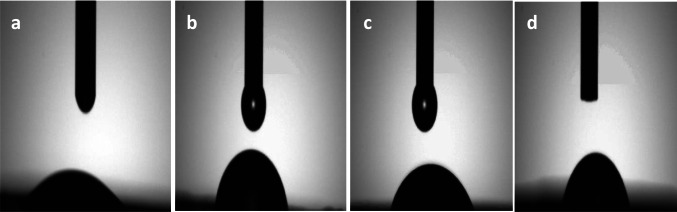
Contact angle of M (**a**), MB15 (**b**), MA15 (**c**), and MC15 (**d**)

The surface wettability was expressed by contact angle measurement. The contact angle for M, MB15, MA15, and MC15 was 26.2°, 62.1°, 52.3°, and 53° respectively. The shape of the water droplet is shown in Fig. 12. The water droplet on the surface of magnetite was easily penetrated due to its high hydrophilicity. The addition of hydrophobic natural products to magnetite improves the hydrophobic properties. The prepared adsorbents were sprayed homogeneously distributed on the surface; they have selective sorption of oil on the water surface, and the oil quickly absorbed into their surfaces; they keep on floating and do not settle down. The wettability of magnetic was decreased with an increase of hydrophobic materials.

### Time impact on removal efficiency of waste oil

**Fig. 13:**
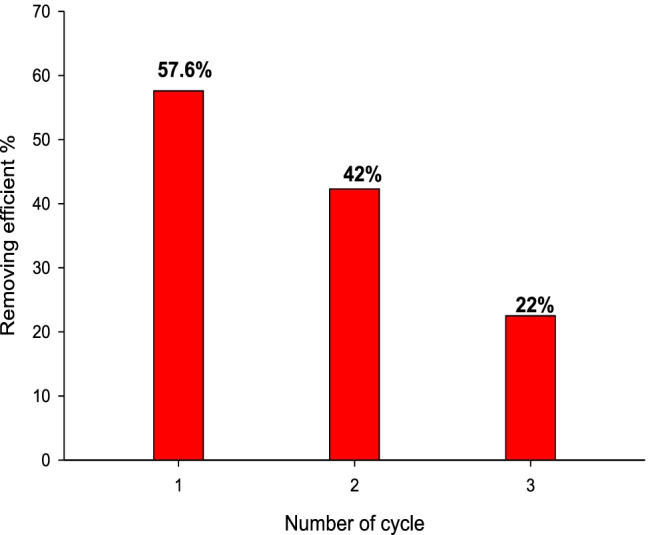
Removal efficiency cycles of MB20

The contact time was affected on the oil removal efficiency; it expresses the kinetics and time equilibrium of adsorption. MB20 was tested by varying the time from 1 to 7 min, with an equal interval (minute) at 25 °C. The removal efficiency was 28, 37, 49, 58, 57, and 65.5% at 1, 2, 3, 4, 5, 6, and 7 min, respectively. The removal efficiency of waste oil increases with an increase in contact time. Initially, the oil adsorption capacity of the prepared adsorbents enhanced with an enhanced contact time due to the presence of active pores on the surface of the adsorbent (Anisi et al. 2021). Then, the adsorbent pores were partially blocked, so the rate of adsorption and diffusion became low. Finally, there is no adsorption or the equilibrium is attained. The maximum removal efficiency was at 4 min contact angle at 25 °C as shown in Fig. 13.

### Temperature impact on removal efficiency

**Fig. 14:**
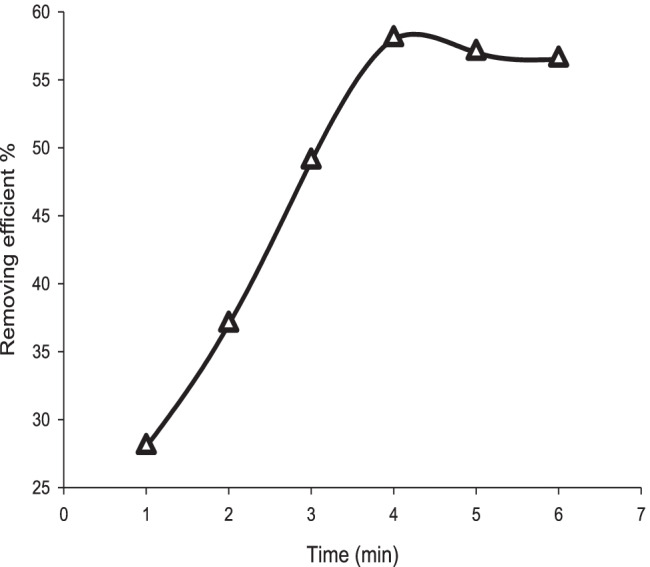
Time impact on removal efficiency of MB20

MB20 was selected depending on its maximum removal efficiency. The temperature plays an important role in the oil removal efficiency, pour point of waste oil, and oil adsorption processes. The temperature’s impact was studied on MB20 as shown in Fig. 14. The removal efficiency was 58, 43, 32, and 25% at 25, 30, 35, and 40 °C. The adsorption process at the liquid–solid interface was decreased with an increase in the temperature due to a decrease in the viscosity or an increase in the rate of diffusion of adsorbed waste oil on the surface of adsorbents. Furthermore, the internal structure of the adsorbent was swollen which facilitates the entry of waste oil molecules into the adsorbent’s pores, so the adsorption capacity was increased (Attallah et al. 2013). So, the optimum oil removal efficiency is at 25 °C. The melting point of rosin is between 140 and 160 °C. The melting point of boswellic acid is between 200 and 270 °C. The melting point of chitosan of about 290 °C depended on the degree of deacetylation. So, the thermal stability of adsorbent with chitosan is more than boswellic acid more than rosin.

### Adsorbent reusability and oil recovery

**Fig. 15:**
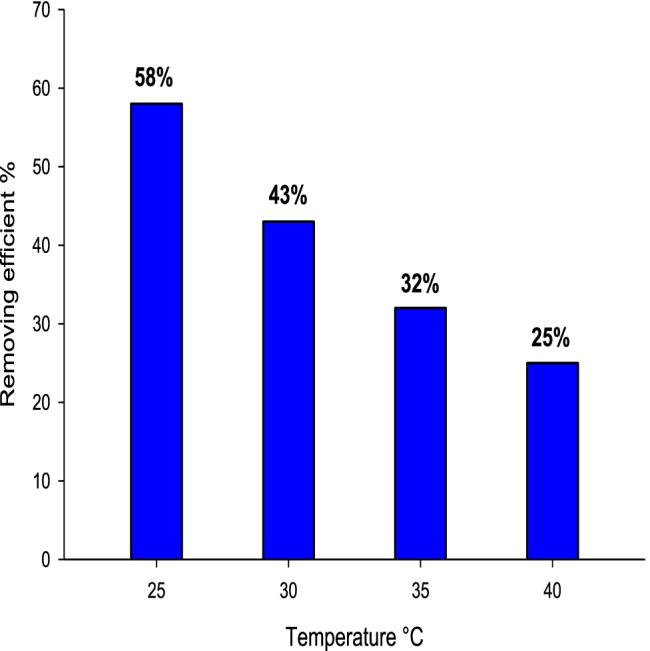
Temperature impact on removal efficiency of MB20

Reusing adsorbents and recovery of waste oil were performed to add value to adsorbents and petroleum oil. The oil recovery and reusability positively impact the environment and economy. The prepared adsorbents can recycle. MB20 has a high value of oil adsorption capacity. The recycle test for MB20 is shown in Fig. 15. At 25 °C, the oil adsorption capacity was lowered to 42 and 22% after the first application. Up to 50% of oil waste was recovered through the adsorption in the first application. The oil adsorption capacity was decreased due to the effect of temperature, washing with a solvent, and drying during the remediation process.

### Production costs

All the used materials are natural products with simple costs, such as rosin, frankincense, and treated shrimp shells (waste). The oil adsorbent’s price changes between 0.27 and US$0.7/L. The prepared adsorbents are easily and simply synthesized with low electricity consumption and working hours. On the other hand, the cost of recovered oil decreases the total cost of the clean-up process.

## Conclusions

The addition of natural products (NP) such as boswellic, abietic acid, and chitosan with different concentrations of 10, 15, and 20% was effectively prepared by the casting process to produce eco-friendly oil nano-adsorbents. During the preparation, a small percentage of magnetite was exposed to oxidation without change in its structure. There is a slight effect on the magnetization behavior of adsorbents due to the addition of NP to magnetite. The contact angle analysis illustrates the hydrophobic property was improved. Depending on the charge of each additional NP, all produced adsorbents have a different surface charge than magnetite. Chitosan nanoparticles adsorb at the outer surface of magnetite, while boswellic and abietic absorb in the bulk of magnetite. Generally, the oil REF was increased with increased amount of added NP. MB20 has a higher value of oil REF and ODC than MA20 and MC20. The preferred contact time is 4 min to achieve high oil REF at 25 °C. High oil REF has occurred at low temperatures. The production cost of all prepared adsorbents is low with a high ability to recycle many times.

## Data Availability

All data and materials for this study are included in the manuscript.
